# Genotypic and allelic frequency of a mutation in the *NHEJ1* gene associated with collie eye anomaly in dogs in Italy

**DOI:** 10.1002/vro2.26

**Published:** 2022-01-29

**Authors:** Stefano P. Marelli, Rita Rizzi, Alessandra Paganelli, Mara Bagardi, Giulietta Minozzi, Paola G. Brambilla, Michele Polli

**Affiliations:** ^1^ Department of Veterinary Medicine University of Milan Lodi Italy; ^2^ Vetogene ‐ ENCI Servizi Milano Italy

## Abstract

**Background:**

A 7.8‐kb deletion in intron 4 of the *NHEJ1* canine gene is associated with Collie Eye Anomaly (CEA). This deletion has been described in sheep‐herding breeds related to the collie lineage and in several other dog breeds. A genetic test based on this association can distinguish three genotypes: normal, carrier and affected. The present study is a retrospective investigation of the presence of the CEA allele frequencies in selected breeds from the Italian dog population over a 10‐year time span.

**Methods:**

Genotype data, for the 7.8 kb deletion in intron 4 of the *NHEJ1* gene, from 496 dogs belonging to Border collie (BC, n = 334), Shetland Sheepdog (SS, n = 74), Australian Shepherd (AS, n = 52), Nova Scotia Duck Tolling Retriever (NS, n = 20) and Rough Collie (RC, n = 16) were analysed. The genetic frequency of CEA allele was estimated in breeds with higher observations (BC, SS and AS).

**Results:**

Healthy carriers were 50%, 45%, 29.6%, 17.3% and 12.5% in SS, NS, BC, AS and RC, respectively. The affected recessive homozygotes were 81.3%, 10.8% and 1.5% in RC, SS and BC, respectively. The CEA allelic frequencies were 0.36, 0.16 and 0.087 in SS, BC and AS, respectively.

**Conclusion:**

The results support the usefulness of this type of genetic analysis to optimize the care of dogs where the CEA mutation is present, including assessing the health risk to susceptible dogs within a breed and to provide an objective basis for breeding programmes.

## 1 INTRODUCTION

Collie Eye Anomaly (CEA) is a hereditary oculopathy affecting the development of the choroid and sclera. British sheep‐herding breeds and their descendants are reported to frequently carry the mutation of the *NHEJ1* gene associated with CEA.^1^ The CEA mutation is also recorded in several other breeds.^2^ Worldwide it is reported that CEA in collie breeds is the most common inherited retinal disease (70‐90%).^1^

The mode of inheritance for CEA is autosomal recessive with incomplete penetrance.^2^ A 7.8‐kb deletion in intron 4 of the *NHEJ1* canine gene is associated with CEA. The clinical phenotype varies considerably: many dogs exhibit no obvious clinical signs and have normal vision throughout life, whereas other phenotypes that are defined as “severely affected” can develop secondary intraocular haemorrhage, retinal detachment and blindness. CEA may be associated with several, more evident anomalies in the eye. Microphthalmia presents with eyeballs that are reduced in size and functionality. Enophthalmia presents as eyeballs that are placed deep into the eye sockets. Mineralization in the cornea is frequently observed and leads to cloudiness. In more advanced stages of the disease, there may be a defect in some structures of the eye called coloboma. In the final stages of the disease detachment of the retina with blindness may occur. CEA is considered homologous to macular coloboma in humans. Comparative analysis of the NHEJ1 region in the genomes of dog, human, mouse and rat have demonstrated conserved binding sites for several DNA‐binding proteins in this location.^3^ Through the genetic test, it is possible to identify CEA affected dogs, recessive homozygotes (carriers) and clinically healthy dogs.^3^

The primary aim of the present study was a retrospective investigation of the presence of the CEA mutated gene in the Italian dog population over a 10‐year period (2010‐2019). Secondary aims including recording the age at which testing was performed and to assess the frequency of the CEA mutation in selected breeds. To the best of the authors’ knowledge, it is the first survey about breed‐specific distribution of the CEA mutation in Italy.

Analysis of the CEA status of the selected breeds, particularly those used for sheep farming, is important to help reduce the prevalence of the genetically affected recessive homozygotes subjects and the clinically healthy carriers.

## 2 MATERIALS AND METHODS

Genotype data for 7.8 kb deletion in intron 4 of the *NHEJ1* gene for 496 dogs belonging to Border collie (BC; 191 females, 143 males; 12% of the registered Italian population), Shetland sheepdog (SS; 41 females, 33 males; 30% of the registered Italian population), Australian Shepherd (AS; 36 females, 16 males; 2% of the registered Italian population), Nova Scotia duck tolling retriever (NS; 10 females, 10 males; 34% of the registered Italian population) and rough collie (RC; 11 females, five males; 4% of the registered Italian population) breeds were collected.

The data used in this retrospective analysis were obtained by the Vetogene Lab, one of the official reference laboratories for the Italian Kennel Club ENCI (FCI associate) and were referred to all dogs genotyped at *NHEJ1* locus for CEA from 1 January, 2010 to 31 March, 2019, as part of a genetic improvement and safeguard programme for dog breeds bred in Italy. Blood samples were collected from the cephalic vein and were sent for the genetic test analysis by veterinarians who officially certified the identification of the sample through microchip control.

The DNA extraction was obtained from 100 to 200 μL of whole blood samples using the commercial kit Qiagen DNeasy Blood & Tissue kit (Qiagen, Hilden, Germany). The presence of the 7.8‐kb deletion (37:28,697,542 ‐ 28,705,340) in the *NHEJ1* gene (Entrez Gene ID 610570) was tested by PCR, in Vetogene Lab, using primers as previously described.^3^

Analyses were conducted using the SAS 9.4 software (SAS Inc., Cary, NC, USA). First, frequencies relative to healthy, carrier, and affected dogs in the available breeds were described. Differences in age at test among breeds were analyzed by Kruskall‐Wallis test using the PROC NPAR1WAY. As a second step, dogs from breeds where more than 20 dogs were sampled were considered. Annual proportions of healthy, carrier, and affected dogs for each breed were calculated and genetic frequencies were estimated. For these analyses, PROC FREQ and PROC ALLELE procedures of SAS were used.

## 3 RESULTS

The healthy carriers were 50.0%, 45.0%, 29.6%, 17.3%, and 12.5% in SS, NS, BC, AS and RC, respectively. The affected recessive homozygotes were 81.3%, 10.8% and 1.5% in RC, SS and BC, respectively; no affected dogs were found in AS and NS (Table 1).

**TABLE 1:** Frequencies of Collie eye anomaly genotypes in five dog breeds included in the study

Genotype	Affected		Normal		Carrier	
Breed	N	Percentage (%)	N	Percentage (%)	N	Percentage (%)
Australian shepherd	0	0.00	43	82.69	9	17.31
Border collie	5	1.50	230	68.86	99	29.64
Nova Scotia duck tolling retriever	0	0.00	11	55.00	9	45.00
Rough collie	13	81.25	1	6.25	2	12.50
Shetland sheepdog	8	10.81	29	39.19	37	50.00
Total	26	5.24	314	63.31	156	31.45

**FIGURE 1:**
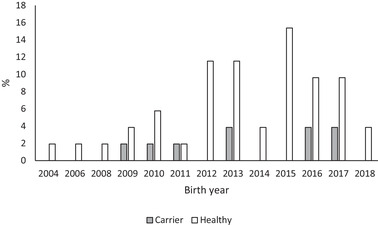
Genotypes (affected, carrier, healthy) at the *NHEJ1* locus separately for birth year for Australian shepherd, n = 52

**FIGURE 2:**
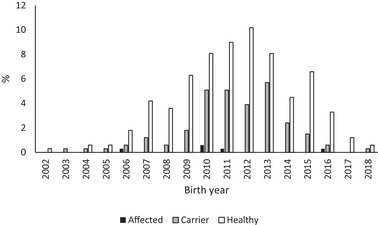
Genotypes (affected, carrier, healthy) at the *NHEJ1* locus separately for birth year for Border collie, n = 334

**FIGURE 3:**
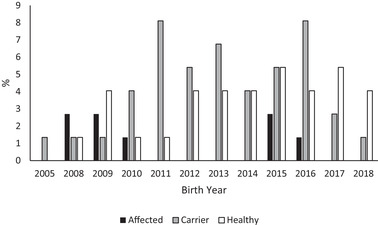
Genotypes (affected, carrier, healthy) at the *NHEJ1* locus separately for birth year in Shetland sheepdog, n = 74

In the breeds with numbers > 20 (AS, BC, SS) the trend in the proportion of carriers for AS and BC showed a decrease towards the end the study period (Figures 1, 2 and 3). In the SS breed, the presence of carriers varied and peaked in the years 2011, 2013 and 2016 (Figure 3).

**FIGURE 4:**
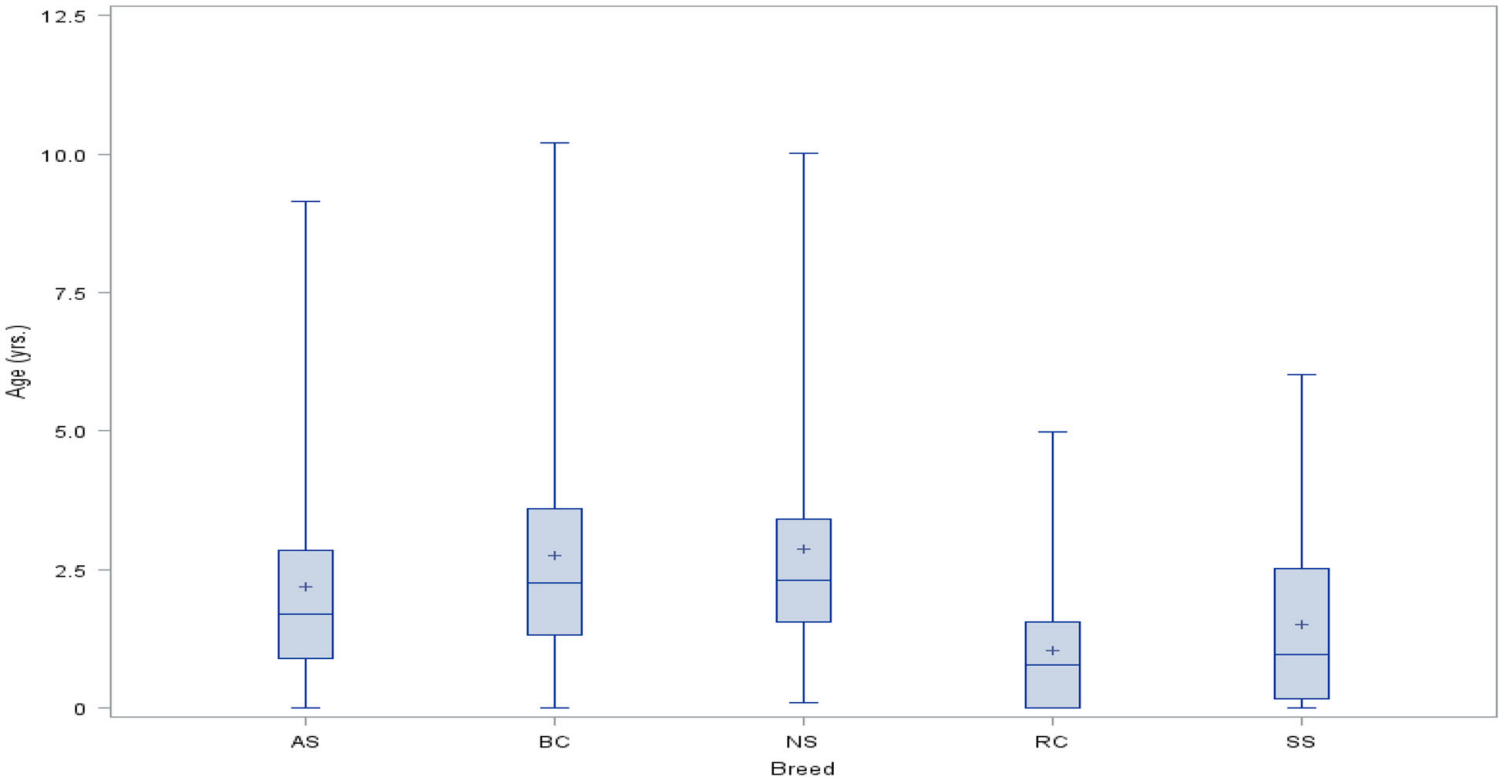
Box‐plot of age (years) at genotyping in five dog breeds. AS: Australian shepherd; BC: Border collie; NS: Nova Scotia duck tolling retriever; RC: rough collie; SS: Shetland sheepdog

For the whole study population, the blood samples were collected at an overall age of 2.44 ±1.95 years. The average age at which NS, BC, AS dogs were genotyped was 2.9 ± 2.4, 2.7 ± 2.0, and 2.2 ± 1.8 years, respectively and was statistically different (p < 0.05) from that recorded for SS (1.5 ± 1.5 years) and RC (1.1 ± 1.2 years). The age range was wide especially for the AS, BC and NS breeds, and some dogs were over 9 years of age when tested (Figure 4).

Gene frequencies are reported in Table 2. Excluding the NS and RC breeds that had <20 dogs tested, it was found that the SS breed showed the highest frequency of the mutated allele (0.36), followed by the BC (0.16) and AS (0.09) breeds.

**TABLE 2:** Collie eye anomaly gene frequency and 95% confidence interval (CI) in three dog breeds where more than 20 dogs were sampled and tested

	Mutated allele		Normal	
	Frequency	CI	Frequency	CI
Australian shepherd	0.086	0.040–0.150	0.913	0.850–0.960
Border collie	0.163	0.135–0.192	0.837	0.808–0.864
Shetland sheepdog	0.358	0.281–0.431	0.642	0.568–0.719

## 4 DISCUSSION

In our study, the BC and AS breeds were the most common sheep‐herding breeds recorded in Italy, at least given that they had the largest number of animals registered with the Italian Kennel Club studbook. The study population, for 2019, comprised BC 46.7%, AS 41.2%, and all of the other breeds comprised 12.1%.^4^

In BC, a relatively high percentage of carriers (29.64%) was detected, compared to very few affected subjects (1.5%). Our data are in accordance with those reported in the Czech Republic (33.8% of carriers and 2% of affected).^5^ In two surveys a lower percentage (equal to 9% and 1.05%) of affected BCs were respectively reported in Switzerland^6^ and Belgium.^7^ It is important to notice that compared to the annual numbers of animals registered every year, BC represents one of the most controlled breeds, with breeding choices aimed to exclude carriers and affected subjects through DNA testing. The BC is the most popular breed that is sampled and submitted for CEA analysis in Italy.

In AS, no affected subjects were detected in our study; this is similar to results reported for the Czech^5^ and Belgian^7^ AS population. However, in the present study AS carriers represented 17.31% of the tested population and this percentage is higher than the one (6.25%) reported for Belgium.^7^ There has been a substantial increase in registrations for the Australian Shepherd breed in the recent years in Italy, from 802 in 2011 to 2567 in 2019;^4^ this was associated with the breeders recommending and arranging genetic testing with the expansion of the breed population.

Regarding the SS breed, our results are in accordance with those reported in the Czech^5^ and Swiss^6^ dog populations. In fact, 10.81%, 15.1% and 16.3% of affected dogs were respectively found in Italian, Czech and Swiss populations. The consistent proportion of carriers discovered (50%) is very close to 53.2% as reported for a Czech SS breed study.^5^

In NS dogs, no affected subjects were found, whereas a high percentage (45%) of carriers was detected. In the same retriever breed, 34.9% of carriers were described in the Czech Republic.^5^ The same authors reported a percentage of 7% of affected subjects in the tested population. The data reported on this breed in Switzerland shows some peculiarities, because no carriers or affected individuals were found.^6^ As the SS and NS had very small population sizes (247 and 58 entries in 2019, respectively)^4^ it is possible to suppose a high risk of inbreeding, a condition which could dangerously limit genetic variation and breeders’ choices and that could be the cause of the remarkable level of carriers found.

The situation of the RC breed is a concern given the small population size and the consequent potential use of a small number of breeding animals which could increase the risk of inbreeding and lead to an increase in the prevalence of the CEA condition.

The high number of carriers found in the BC (29.64%), SS (45%) and NS (50%) breeds are probably linked to the objective of breeders to recognize heterozygous dogs with greater interest than homozygotes, the latter being clinically identifiable in the first six weeks of lif^2^ and therefore often not subjected to genetic analysis.^2^

The trend of reduction in the proportion of carriers in the AS and BC breeds shows the effectiveness of an accurate training and information campaign for breeders of the two breeds carried out through press releases, seminars and conferences by the Italian Kennel Club ENCI. The ENCI wanted to promote dog health and welfare through encouraging genetic testing by breeders in order to reduce the proportion of carriers in the AS and BC breeds.

Given the high proportion of affected dogs in RC and of carriers in the SS breeds, it is important that DNA testing at an early age is undertaken by breeders. It should be noted that in Switzerland 53.8% of RC dogs under the age of eight months were genotyped at *NHEJ1* locus; that in the SS breed in the same age group the percentage tested was lower at 18.7%.^6^ Unfortunately, the average age at DNA test found in all the examined breeds in our study was rather late in relation to the possibility of limiting the spread of the disease (2.44 ± 1.95). Given the reproductive capability of dogs at two years of age this could lead to matings of uncontrolled pairs. It is, therefore, important to clearly communicate to breeders the need to have DNA testing performed as early as possible in the life of future sires and dams. At the same time, it should be remembered the usefulness of the ophthalmological examination of puppies before eight weeks of age as an efficient method to identify dogs with clinical signs of CEA.^8^

From the evaluation of the allele frequencies relating to the mutated allele for CEA, in the AS we found a frequency of 8.65%, while lower frequencies were reported in the Czech Republic (4.5%) and in Belgium (3.1%).^5^, ^7^ From the results obtained, it appears that the Italian situation is quite similar to data from the Czech Republic^5^ and Japan.^9^ It should be noted that dogs bred in Belgium have a lower gene frequency.

Our findings describe the frequencies of the CEA mutation in Italy over a 10‐year time span, a clear interest in DNA test has been demonstrated; moreover, a reduction of the age at the test would be ideal. Population sizes, number of breeders per breed and breeding strategies, can all influence the possibility to improve population fitness through biodiversity and selective matings aimed to improve dog health and welfare. Testing for the CEA mutation is an effective tool in population management. The use of DNA testing at an early age for mendelian recessive conditions such as CEA should be considered an important target for breeders, breed associations and national clubs.

## CONFLICTS OF INTEREST

The authors declare they have no conflicts of interest.

## ETHICAL APPROVAL

All animals that were recruited for this study were client‐owned; the owners provided written consent. Blood samples were collected by an authorized veterinarian.
